# Genome-Wide Association Study Revealed Putative SNPs and Candidate Genes Associated with Growth and Meat Traits in Japanese Quail

**DOI:** 10.3390/genes15030294

**Published:** 2024-02-25

**Authors:** Natalia A. Volkova, Michael N. Romanov, Alexandra S. Abdelmanova, Polina V. Larionova, Nadezhda Yu. German, Anastasia N. Vetokh, Alexey V. Shakhin, Ludmila A. Volkova, Alexander A. Sermyagin, Dmitry V. Anshakov, Vladimir I. Fisinin, Darren K. Griffin, Johann Sölkner, Gottfried Brem, John C. McEwan, Rudiger Brauning, Natalia A. Zinovieva

**Affiliations:** 1 L. K. Ernst Federal Research Center for Animal Husbandry, Dubrovitsy, Podolsk 142132, Moscow Oblast, Russia; natavolkova@inbox.ru (N.A.V.); abdelmanova@vij.ru (A.S.A.); volpolina@mail.ru (P.V.L.); ngerman9@gmail.com (N.Y.G.); anastezuya@mail.ru (A.N.V.); alexshahin@mail.ru (A.V.S.); ludavolkova@inbox.ru (L.A.V.); alex_sermyagin85@mail.ru (A.A.S.); 2 School of Biosciences, University of Kent, Canterbury CT2 7NJ, Kent, UK; d.k.griffin@kent.ac.uk; 3 Breeding and Genetic Center “Zagorsk Experimental Breeding Farm”—Branch of the Federal Research Center “All-Russian Poultry Research and Technological Institute”, Russian Academy of Sciences, Sergiev Posad 141311, Moscow Oblast, Russia; a89265594669@rambler.ru; 4 Federal Research Center “All-Russian Poultry Research and Technological Institute” of the Russian Academy of Sciences, Sergiev Posad 141311, Moscow Oblast, Russia; olga@vnitip.ru; 5 Institute of Livestock Sciences (NUWI), University of Natural Resources and Life Sciences Vienna, 1180 Vienna, Austria; johann.soelkner@boku.ac.at; 6 Institute of Animal Breeding and Genetics, University of Veterinary Medicine, 1210 Vienna, Austria; gottfried.brem@agrobiogen.de; 7 AgResearch, Invermay Agricultural Centre, Mosgiel 9053, New Zealand; john.mcewan@agresearch.co.nz (J.C.M.); rudiger.brauning@agresearch.co.nz (R.B.)

**Keywords:** GWAS, genotyping by sequencing, Japanese quail, SNPs, candidate genes, body weight, meat performance traits

## Abstract

The search for SNPs and candidate genes that determine the manifestation of major selected traits is one crucial objective for genomic selection aimed at increasing poultry production efficiency. Here, we report a genome-wide association study (GWAS) for traits characterizing meat performance in the domestic quail. A total of 146 males from an F_2_ reference population resulting from crossing a fast (Japanese) and a slow (Texas White) growing breed were examined. Using the genotyping-by-sequencing technique, genomic data were obtained for 115,743 SNPs (92,618 SNPs after quality control) that were employed in this GWAS. The results identified significant SNPs associated with the following traits at 8 weeks of age: body weight (nine SNPs), daily body weight gain (eight SNPs), dressed weight (33 SNPs), and weights of breast (18 SNPs), thigh (eight SNPs), and drumstick (three SNPs). Also, 12 SNPs and five candidate genes (*GNAL*, *DNAJC6*, *LEPR*, *SPAG9*, and *SLC27A4*) shared associations with three or more traits. These findings are consistent with the understanding of the genetic complexity of body weight-related traits in quail. The identified SNPs and genes can be used in effective quail breeding as molecular genetic markers for growth and meat characteristics for the purpose of genetic improvement.

## 1. Introduction

**Figure 1:**
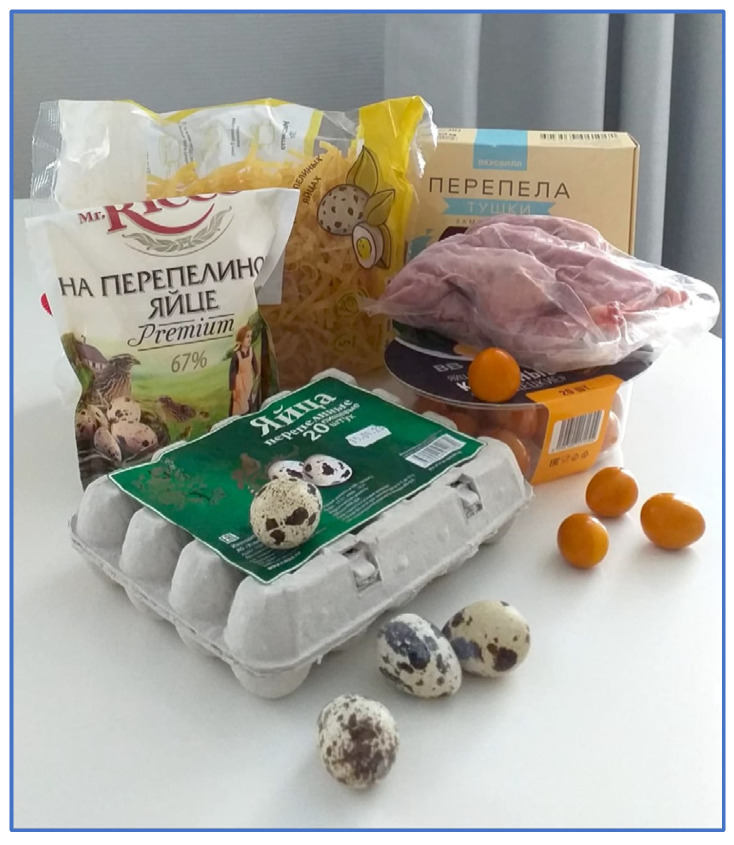
Assortment of quail products: wholesale commercial egg tray and a pack of seasoned smoked hard-boiled eggs (in the foreground); whole egg mayonnaise, quail egg nutrient noodles, and frozen quail carcasses (in the background). Credit: authors’ own photo.

Japanese quail [*Coturnix japonica* (CJA) Temminck and Schlegel, 1848] products (Figure 1) occupy a strong position in the overall market assortment of food products provided by the livestock industry. Both quail eggs and meat are in great demand among the worldwide population [1,2,3,4,5]. Quail meat is a dietary product characterized by high nutritional value and good taste. Quail meat contains a small amount of fat (3–4%) and a significant proportion of proteins (20%). In terms of vitamin and mineral content, quail meat is superior to the meat of broiler chickens [6,7,8,9].

In recent years, there has been an increase in the production of quail products due to growing consumer demand for quail eggs and meat and the high profitability and commercial attractiveness of this industry. The main advantages of using domestic quails for meat production include their rapid growth, early maturation, and adaptability to various environmental conditions (e.g., [10,11]). The period of embryonic development in quails is 17–18 days, and sexual maturity occurs at the age of 35–45 days [7,12,13,14,15]. These biological characteristics of quails provide a short generation interval; thus, a large population of birds can be obtained in a relatively short period of time. From the viewpoint of the commercial quail meat production, the high resistance of quail to infectious diseases is also beneficial [1,7,12,16]. This reduces the cost of veterinary protection and care, makes it possible to obtain environmentally friendly products without the use of antibiotics, and requires less vaccination [17].

The basis for sustainable development and competitiveness of meat quail farming is the creation and use of highly productive breeds and crosses. This depends on effective genetic breeding aimed at seeking and detecting valuable genotypes using cutting-edge methodologies to tackle molecular genetic mechanisms of the formation and manifestation of major selected traits. To date, biomedicine and biotechnology information databases, e.g., the National Center for Biotechnology Information (NCBI) [18], have been created as resources containing a large amount of data on quantitative trait loci (QTLs) and candidate genes associated with economically important traits in farm animals [19,20]. In poultry, a significant proportion of studies have been carried out on chickens using both microsatellites (e.g., [21,22,23,24]) and SNPs (e.g., [25,26,27]) as genetic markers. For quail, these studies have also been conducted but are much less advanced (e.g., [28]). A number of studies have reported the relationship between microsatellite markers and indicators of quail growth [29,30,31], development [32], meat quality [33], and egg productivity [34]. Among the disadvantages of this microsatellite-assisted approach, certain complications in mapping and identifying the respective genes should be noted. Over the last years, there has been a rise in the number of studies that employ SNPs as genetic markers and genome-wide SNP genotyping [10,35]. Using this technique and the genome-wide association study (GWAS) approach in quail, significant SNPs and putative genes have been identified in terms of their association with performance traits [36,37], behavior [38,39], plumage color [10], reproductive traits [40], and egg quality [41,42]. The lack of commercial SNP arrays for Japanese quail makes it difficult to search for SNPs and identify genes associated with traits important for breeding, suggesting other SNP genotyping approaches [43]. Recently, we applied, for the first time, genotyping-by-sequencing (GBS) technology [44,45] in Japanese quails to generate multiple genome-wide SNP markers for executing GWAS of growth dynamics [43] and characterizing within- and between-breed genetic diversity [46].

**Figure 2:**
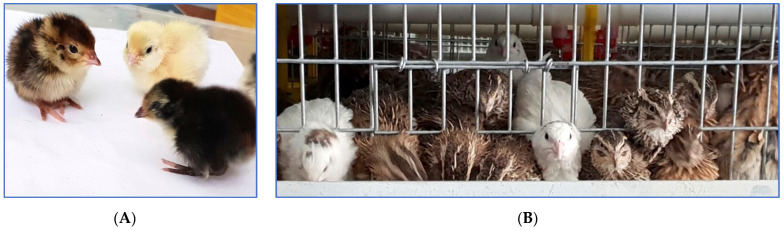
Quails of the F_2_ reference population: (**A**) at 3 days of age and (**B**) at 7 weeks of age.

In the current study, we focused on searching for and identifying SNPs associated with meat performance traits in domestic quail, such as body weight at different ages (BW), average daily body weight gain (ADBWG), dressed carcass weight (DCW) and weights of components, namely, breast (BrW), thigh (TW), and drumstick (DW). In accordance with this goal, a GWAS of meat quality traits was conducted based on GBS data. For this purpose, we utilized an F_2_ reference quail population (Figure 2) generated via crossbreeding between the divergent Texas White (TEW; fast growing) and Japanese (JAP; slow growing) breeds, i.e., the approach that is conventionally used for genetic dissection of quantitative traits (e.g., [22,30,31,32,47,48]).

## 2. Materials and Methods

### 2.1. Experimental Birds

Fertile quail eggs were purchased from Genofond LLC (All-Russian Poultry Research and Technological Institute, Sergiev Posad, Russia; [49]). Birds were raised at the L. K. Ernst Federal Research Centre for Animal Husbandry, and their DNA samples were obtained. The vivarium premises for keeping quails were equipped for a full rearing and production cycle, from young animals to adult birds. In these premises, the temperature was maintained from 20 to 25 °C and humidity from 55 to 65%, depending on the season. Moderate fluorescent lighting was used, with a brightness in the feeder area of no more than 50 lux. At all stages of rearing, the birds had free access to commercial feed and clean running water. The nutritional value of the feed was 2800–2850 kcal, with no less than 23% crude protein for young animals, and 2900 kcal, with no less than 18% crude protein for quails older than 6 weeks of age.

**Figure 3:**
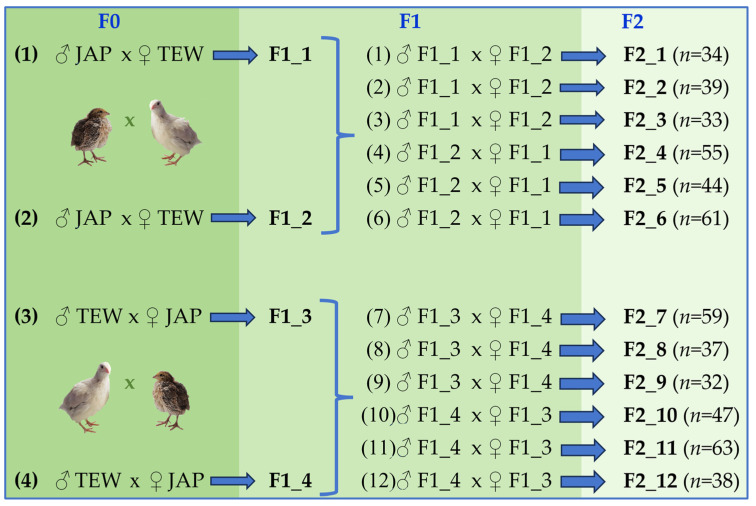
Overview of the experimental design. The reference population consisting of 146 F_2_ male quails was created and extensively phenotyped using reciprocal crosses between a slow-growing Japanese and a fast-growing Texas White breed. *n*, number of progenies (of both sexes) from each F_2_ family.

This study assessed a total of 146 males of an F_2_ reference population (Figure 2) produced by crossing the following two parent breeds [43]: (1) JAP, a slow-growing breed of layer (lighter) type, and (2) TEW, a fast-growing breed of meat (heavier) type (Table 1). At the first stage, four families were formed, each of which included one male and five females of the original breeds, JAP and TEW, to perform their reciprocal crosses according to the experimental design presented in Figure 3. Individuals of the grandparent stock were not related and were mated to produce the following families: F0_1 and F0_2 (each included one JAP male and five TEW females) and F0_3 and F0_4 (each consisted of one TEW male and five JAP females). From each family, 20–30 F_1_ progenies were obtained and used to generate the F_2_ reference population. For this purpose, we selected 12 F_1_ families (F1_1 to F1_12) consisting of one male and three females that were not close relatives. As a result of mating these F_1_ individuals, F_2_ offspring were produced (groups F2_1 to F2_12, *n* = 542), and 146 F_2_ males were chosen for further research (groups F2_1 to F2_6, *n* = 72; and F2_7 to F2_12, *n* = 74). Thus, the original parent breeds made an equal contribution to the formation of the experimental population of F_2_ quails used in this study for the GWAS analysis of meat traits.

When forming the experimental sample, the phenotypic characteristics of F_2_ quails were taken into account. In each F_2_ group, 8–14 individuals were selected with contrasting values (maximum, minimum) of the studied traits (as described in Section 2.2). Based on the ranking results of the experimental F_2_ birds according to the established meat productivity indicators, a more pronounced scatter of indicators was observed in males as compared to females in most of the studied F_2_ groups. Because of this and in order to increase the power of detecting QTLs as well as to exclude the effects of the sex factor, it was decided to use males for further analysis.

### 2.2. Phenotypic Traits

F_2_ males were phenotyped for seven growth and meat traits as follows: BW, ADBWG, DCW, BrW, TW, and DW. Using laboratory scales, BW was measured at the beginning and end of the experiment, i.e., at 1 (BW1) and 56 (BW56) days of age. Based on the BW data obtained, ADBWG was computed for the whole growing period from 1 to 56 days. At the age of 56 days, birds were euthanized for experimental weight assessment of the carcass and its components. The carcasses were cut into the following parts: breast, thighs, and drumsticks, followed by separating the muscles, bones, and skin. For the purpose of this investigation, the weights of thighs (TW) and drumsticks (DW) were determined before separating the skin and bones, with mean values being calculated for the left and right thigh (drumstick) obtained from each animal. The sample of examined birds met the criterion of normal distribution for the studied meat traits.

### 2.3. Sampling and DNA Extraction

Feather pulp samples were collected from 146 F_2_ male quails. A Syntol kit for animal tissue (Syntol, Moscow, Russia) was used to isolate DNA. A Qubit 3.0 fluorimeter (Thermo Fisher Scientific, Wilmington, DE, USA) was used to measure the concentration of DNA solutions. Thermo Fisher Scientific’s NanoDrop-2000 instrument was used to measure the OD260/280 ratio in order to evaluate the purity of the extracted DNA.

### 2.4. Sequencing, Genotyping and SNP Quality Control

GBS analysis [44] was performed to genotype quails, and the primary procedures were library preparation, sequencing quality control (QC), SNP identification, and the creation of a genomic relationship matrix. The GBS libraries were created specifically using the techniques published in Elshire et al. [44] with modifications made according to Dodds et al. [58]. One GBS library was constructed using a *Pst*I–*Msp*I double digest and contained negative control samples lacking DNA. Pippin Prep (SAGE Science, Beverly, MA, USA) was used to select fragments from libraries that ranged in size from 220–340 bp (genomic sequence plus 148-bp adapters). We employed a set of 768 adapter sequences containing 10 bp barcodes (signified as N in the sequences below) created by Illumina (Illumina, Inc., San Diego, CA, USA) and Integrated DNA Technologies, Inc. (Coralville, IA, USA), which were at least three mutational steps apart from one another. The following were the respective adapter sequences: PstI_barcode_adapter_F, ACACTCTTTCCCTACACGACGCTCTTCCGATCTNNNNNNNNNNTGCA; PstI_barcode_adapter_R, NNNNNNNNNNAGATCGGAAGAGCGTCGTGTAGGGAAAGAGTGT; MspI(Y)_Common_F, CGAGATCGGAAGAGCGGACTTTAAGC; and MspI_Common_R, GTGACTGGAGTTCAGACGTGTGCTCTTCCGATCT. Using v1.5 reagents and an Illumina NovaSeq 6000, single-end sequencing (1 × 101 bp) was carried out. Using a unique QC process called DECONVQC [59,60], raw fastq files were quality tested using FastQC as one of the QC stages [61].

The genome sequence of the Japanese quail (CJA 2.0; [39]) as well as the databases Ensembl 104 and Ensembl Genomes 51 (published on 7 May 2021; [62]) were utilized as reference genomes. Using the cutadapt program, adapters were removed, and the fastq file was demultiplexed (via separation by samples, i.e., obtaining individual fastq files using a list of barcodes) [63,64]. The FastQC software (Version 0.10.1) application was used to perform fastq file QC [61]. The bowtie2 package (Version 2.4.4) [65] was used for reference genome alignment and indexing, while samtools [66] was used to sort bam data.

Post filtering of the raw data, a total of 115,743 SNPs were generated, while there were 92,618 SNPs left after QC that were used for the next analysis. By implementing commands from the snpGBS package [67] and bamtools [66] to create a single multi-sample VCF file (bcftools mpileup … | bcftools call -m - | bcftools view -M2 …), joint genotyping of the output files was performed. Using R software (Version 4.0.0) [68], the data were produced in a file format suitable for further analysis. The PLINK 1.9 program [69] was exploited for the QC of SNP detection. Based on the genotyping efficiency parameter (MAF 0.05), a filter was applied to the quail genotypes, and genotyped SNPs in less than 90% of samples (geno 0.1) were disregarded from the study.

### 2.5. Principal Component Analysis

Principal component analysis (PCA; [70]) was carried out and visualized using the ggplot2 R package [71]. Data files were produced in the R software (Version 4.0.0) environment [72].

### 2.6. Genome-Wide Association Study and Statistical Analyses

To identify associations of SNPs with traits of meat-producing ability, regression analysis was used in PLINK 1.9. The following standard linear regression model was used:

*y* = *μ* + *GenFam_F_* (*cov*) + *b*_1_ *SNP_k_* (*X*) + *e*,

where *y* is the phenotypic value of a trait, *μ* is the average value of a phenotype, *GenFam_j_* (*cov*) is the covariance matrix of the population structure for the corresponding generations of families *F*, individuals, *b*_1_ is the regression coefficient of the effect of the *k*-th SNP in the matrix of genotypes *X*, and *e* is the residual (random) effect models.

The significance of SNP effects and the identification of significant regions in the quail genome were assessed using the test for null hypotheses and Bonferroni corrections. To be able to describe the SNPs identified in the quail genome and provide a sufficiently detailed description of QTLs associated with meat productivity traits, the following two confidence thresholds were selected: *p* < 5.4 × 10^−7^ (Bonferroni) and *p* < 1.0 × 10^−5^ (selected by default in the R package qqman).

The data were visualized in the qqman package using the R programming language (Version 4.0.0) [73].

The search for candidate genes located in the regions of identified SNPs was carried out using the genome assembly CJA 2.0 [39,62].

## 3. Results

### 3.1. Population Stratification

**Figure 4:**
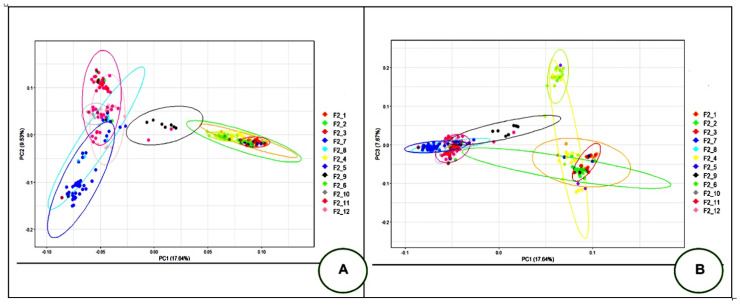
Principal component analysis (PCA) of the F_2_ quail reference population based on GBS data. (**A**) PCA performed in the plane of the first (PC1, *X*-axis) and second (PC2, *Y*-axis) components. (**B**) PCA performed in the plane of the first (PC1, *X*-axis) and second (PC3, *Y*-axis) components. Individuals from different groups are indicated by different colors.

The PCA output revealed the distribution of the studied F_2_ reference population into four main clusters. The first principal component (PC1) was responsible for 17.64% of the genetic variability, and the second principal component (PC2) explained 9.53% of the genetic variability. Herewith, the groups F2_1 to F2_12 were differentiated into three subgroups as follows: (1) F2_1, F2_2, F2_3, F2_4, and F2_6; (2) F2_5, F2_7, F2_8, F2_10, F2_11, and F2_12; and (3) F2_9. Using PC1 and the third principal component (PC3; 7.67% of the genetic variability), these groups were divided into the following three subgroups: (1) F2_7, F2_5, F2_8, F2_9, F2_10, F2_11, and F2_12; (2) F2_1, F2_2, F2_3, and F2_4; and (3) F2_6, the latter being evenly distant from subgroups 1 and 2. This stratification information is presented in Figure 4.

The identified structure of the F_2_ population was determined by the characteristics inherited from the parent breeds, JAP and TEW. In particular, as the initial paternal breed, JAP was used to produce groups F2_1, F2_2, F2_3, F2_4, F2_5, and F2_6, and TEW was used to produce groups F2_7, F2_8, F2_9, F2_10, F2_11, and F2_12. Taking into account that the principal component vectors (PC1, PC2, and PC3) divided the F_2_ quail reference population sample into subgroups with a fairly clear proportion of genetic variations (17.64, 9.53, and 7.67%, respectively), we used four vectors to clarify the population stratification more distinctly (with PC4 = 6.21%; see also the respective PCA plot for the F_2_ quail reference population in Figure S1). Considering the observed stratification (structure) of the F_2_ population, we further performed the GWAS outlined below using the first four PCs as covariates.

### 3.2. Genome-Wide Association Study

Descriptive statistics for the meat productivity traits of male quails from the F_2_ reference population are summarized in Table 2 (see also Supplementary Information) and demonstrated the distribution of phenotypic indicators for the measured meat traits in the studied quails. Accordingly, the variability of the studied characteristic values as assessed by coefficients of variation was established to be within 10–14% as presented in Table 2.

**Figure 5:**
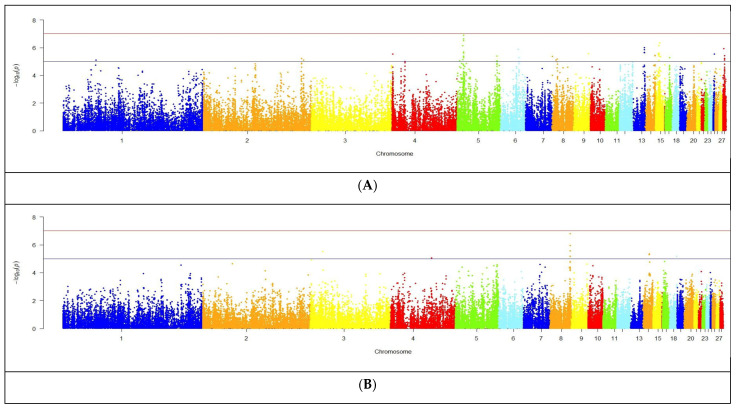

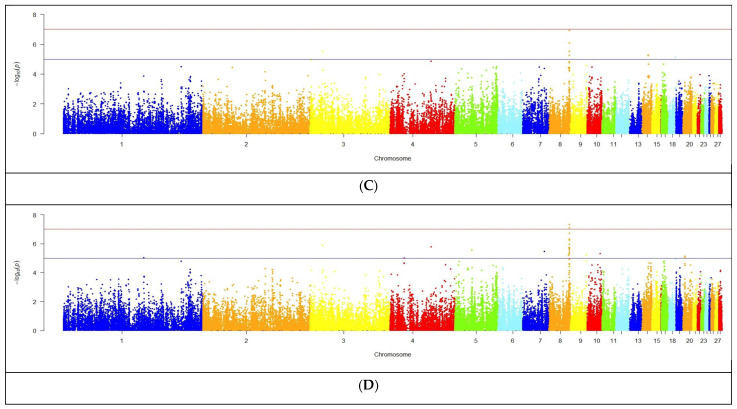

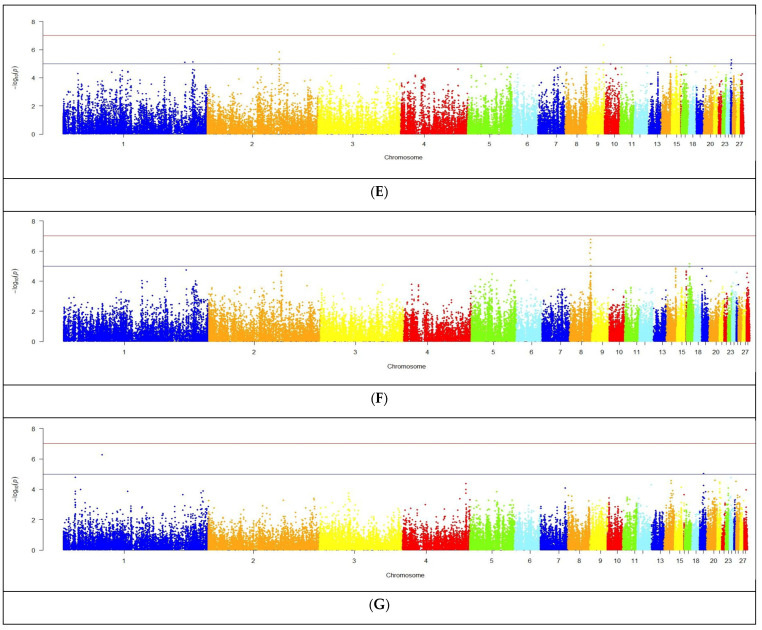
Manhattan plots of the GWAS results for the studied meat production traits: (**A**) body weight at 1 day of age, (**B**) body weight at 56 days of age, (**C**) average daily body weight gain, (**D**) dressed carcass weight, (**E**) breast weight, (**F**) thigh weight, and (**G**) drumstick weight. Distribution of SNPs in quail chromosomes for single traits are shown relative to the thresholds for the genome-wide nominal significance level (−log_10_ (*p*)) according to the estimated probability values of *p* < 1.0 × 10^−5^ (lower line) and *p* < 5.4 × 10^−7^ (upper line). Points are color-coded only to visualize chromosome separation.

The results of the GWAS analysis for meat performance traits in the F_2_ quail reference population are presented in Figure 5. We discovered putative SNPs on chromosomes 1–28 (CJA1–CJA28) that had a high level of significance and were associated with the studied indicators of meat productivity in F_2_ males. The maximum number of identified SNPs was localized on CJA8 (44 SNPs), while the minimum number was observed on CJA 7, 10, 17, 18, and 19 (one to two SNPs). Inspection of the Q–Q plots (Figure S2) revealed that the observed *p*-values resulting from the GWAS for BW56, ADBWG, TW, and DW did not deviate essentially from the expected values, suggesting that the used models for the GWAS were reliable.

Summarized GWAS results for growth and meat traits in male quails of the F_2_ reference population are presented in Table 3 (see also the respective GWAS statistics in Supplementary Information). Accordingly, there were significant associations of BW1 with 58 SNPs localized on CJA 1, 2, 4, 5, 13–15, 17, 25, and 28. The largest number of putative SNPs was observed on CJA5 (20 SNPs), whereas their smallest numbers were on CJA 1, 4, 17, and 25 (one to two SNPs). At the final age of grown quails, the number of identified significant SNPs associated with BW56 was smaller than that for BW1 and amounted to 18 SNPs. These SNPs were located on CJA 1, 2, 3, 4, 8, 12, 14, 17, and 18. Hereby, the maximum number of significant SNPs was found on CJA8 (five SNPs), and the minimum on CJA 1, 3, 4, 12, and 18 (one SNP on each). According to the GWAS analysis for ADBWG in the period from 1 to 56 days, there were eight putative SNPs associated with this indicator and identified on CJA 3, 8, 14, and 18. The maximum numbers of these SNPs for ADBWG as well as for the BW trait were observed on CJA8 (five SNPs).

As far as weight indicators of carcass and its components, the present GWAS revealed a relatively high number of putative SNPs associated with DCW and BrW indicators (33 and 18 SNPs, respectively). For the other studied traits characterizing the weight parameters of the quail carcass parts, the numbers of significant SNPs were eight for TW and two for DW. The maximum numbers of these SNPs were localized on the following chromosomes: CJA8 for DCW (26 SNPs); CJA 9 and 14 for BrW (four to five SNPs); and CJA8 for TW (seven SNPs). The minimum number of SNPs (one to two SNPs) was identified for DCW (on CJA 1, 3–5, 7, 9, and 10), BrW (on CJA 1–3 and 5), and TW trait (on CJA17).

Comparative analysis of GWAS data led to 14 SNPs significantly associated with several F_2_ quail traits assessed in this study. In particular, six putative SNPs were simultaneously identified for three traits, four common SNPs were significantly associated with four traits, and four common SNPs were shared between five traits. For one of the traits studied, DW, no SNPs were found in common with the other traits.

### 3.3. Candidate Genes

With regard to putative SNPs shared by a group of studied traits (i.e., four to five traits), we were able to use them to annotate candidate genes associated with quail meat performance. Structural annotation revealed the following five genes described in the NCBI and Ensembl databases [18,62]: G protein subunit alpha L (*GNAL*), DnaJ heat shock protein family (Hsp40) member C6 (*DNAJC6*), leptin receptor (*LEPR*), sperm associated antigen 9 (*SPAG9*), and solute carrier family 27 member 4 (*SLC27A4*). These genes mapped to four chromosomes as follows: CJA2 (one gene), CJA8 (two genes), CJA17 (one gene), and CJA18 (one gene). The significant SNPs and relevant candidate genes associated with BW56 and meat traits in quail are shown in Table 4 (see also Supplementary Information).

## 4. Discussion

Recently domesticated Japanese quail are valuable as a model avian species for genetic studies and as a remarkable agricultural commodity [10,47]. One of the important prerequisites of genomic selection aimed at improving livestock farming efficiency is the identification and mapping of SNPs and candidate genes (e.g., [74]) that are associated with, and control, the manifestation of phenotypes of interest and selection of relevant quantitative traits using GWAS technology (e.g., [75,76,77]). We reported here a GWAS for meat performance characteristics in male quails of an F_2_ reference population. This population was obtained by crossing two divergently selected quail breeds contrasting in terms of growth rates, with one of them (JAP) being of the layer type and characterized by a relatively low growth rate and the other one (TEW) being of the meat type, with a greater ADBWG and good meat qualities. Thus, the F_2_ quail reference population we developed was a convenient model for conducting this GWAS for meat productivity. This implemented approach was in line with the creation and wide implementation of F_2_ reference populations for molecular genetic studies in various poultry species, e.g., in chickens [22,78], turkeys [79], ducks [48], and quails [35,36,38,41,47]. This enabled an increase in the power and sensitivity of detecting QTLs, when forming a sample of experimental birds for the GWAS analysis (despite a relatively low sample size). Furthermore, in each F_2_ group, individuals with the most contrasting values (maximum, minimum) of the studied characteristics were selected.

The traits of BW, ADBWG, DCW, and carcass components are important meat performance indicators, depending on the parameters of poultry feeding and management (e.g., [80]), and are also genetically determined by many QTLs (e.g., [47]). As shown by Abou-Kassem et al. [8], when compared to female quails, males have the highest meat quality and composition characteristics. Our GWAS revealed the respective putative SNPs associated with such meat traits in male quails as BW1, BW56, ADBWG, DCW, BrW, TW, and DW. From the viewpoint of using SNPs in genomic selection, the search for SNPs and genes simultaneously associated with several productive traits is of special interest. In our study, analysis of GWAS results revealed the presence of 14 SNPs and five genes (*GNAL*, *DNAJC6*, *LEPR*, *SPAG9*, and *SLC27A4*) common to few quail meat traits. Our data were concordant with the idea about the genetic complexity of BW-related traits in Japanese quail (e.g., [47,81]).

With respect to the above five candidate genes established in this study, only one gene (*LEPR*) was previously shown to be associated with growth rate and meat yield traits in quails [82,83,84]. Specifically, Wang et al. [84] studied the *LEPR* gene polymorphism in meat-type quails at the age of 3 and 5 weeks and found *LEPR* SNPs associated with growth indicators and carcass characteristics. These included shank circumference and length, breastbone length, heart rate, whole net carcass rate, chest width, body length, and leg muscle rate. In our study, we discovered a relationship between the *LEPR* gene and such growth parameters as BW56, DCW, BrW, TW, and ADBWG. It should also be noted that a fairly large number of other studies have demonstrated the *LEPR* gene effects on growth, productivity, and reproduction in various farm animals [85,86,87,88,89,90,91], including poultry [78,92,93,94].

Concerning other candidate genes we reported here, there was rather limited information on their effect relevant to the growth and productive qualities in animals, including poultry. For instance, Zhang et al. [95] observed an association of the *GNAL* and *SPAG9* genes with abdominal fat deposition in chickens. Revilla et al. [96] established the influence of the *SLC27A4* gene on pig meat quality, in particular, the lipid composition of carcass lard. Zhang et al. [97] identified SNPs in the *DNAJC6* gene region associated with body weight in Duroc × Landrace × Yorkshire crossbred pigs. Thus, of the five discovered candidate genes for quail meat performance, our data confirmed the known effects of only one gene, i.e., *LEPR*, presenting further compelling evidence in support of a previous suggestion that the *LEPR* gene could be used as a molecular genetic marker to enhance the growth and carcass qualities of quail [84]. For the other putative genes, their association with growth and performance in farm animals (*SLC27A4* and *DNAJC6*) and poultry (*GNAL* and *SPAG9*) has also been reported. Therefore, subsequent detailed research is required to elucidate the impact of these candidate genes on the productive traits in Japanese quail.

Collectively, the present investigation relied on GWAS as a potent tool for promoting marker-assisted selection [98]. Based on such high-throughput genetic markers as SNPs, our findings procured further GWAS-assisted information for their potential applications in quail breeding and enhancing quail meat performance.

## 5. Conclusions

Using the molecular technique of GBS, we detected many thousands of SNPs and employed them for the GWAS-based analysis of economically important indicators characterizing growth and meat production in an F_2_ reference population of Japanese quail. The current study contributed to the identification of SNPs and candidate genes associated with growth performance and carcass quality in quail at 8 weeks of age, suggesting their genetic complexity. The maximum number of identified SNPs was observed on CJA8 (44 SNPs), while the minimum number was revealed on CJA 7, 10, and 17–19 (one to two SNPs). The association findings obtained are essential for effective quail breeding and future genomic selection in this popular poultry species. The identified SNPs can be further used as molecular markers in breeding programs to increase the BW of quails as well as improve other indicators of their meat quality.

## Figures and Tables

**Table 1:** Grandparental quail breeds employed for generating the F_2_ reference population.

Breed	Code	*N* ^1^	Origin	Performance ^2^	References
Japanese 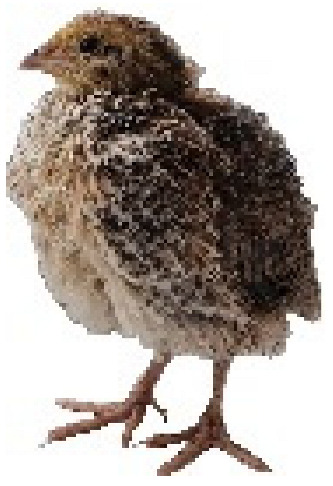	JAP	12	Japan; domesticated in Japan and China as early as the 12th century; selected in the first half of the 20th century, imported to the USSR from Japan in the middle of the 20th century and/or from Yugoslavia in 1964	*Layer type* 6 week body weight: 180 g, females; 150 g, males	[3,49,50,51,52,53,54]
Texas White (or Texas Pharaoh, White Pharaoh, Snowy) 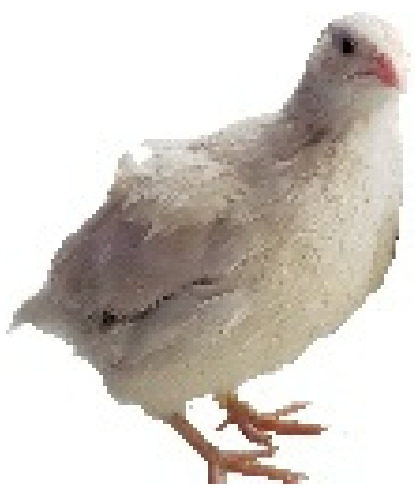	TEW	12	Texas, USA; descended from crossing the Pharaoh and English White breeds	*Meat type* 6 week body weight: 280 g, females; 240 g, males	[53,54,55,56,57]

^1^ *N*, number of grandparental individuals. ^2^ Breed performance data from Roiter et al. [53].

**Table 2:** Descriptive statistics ^1^ for meat productivity indicators of male quails from the F_2_ reference population.

Traits	*n*	Maximum	Minimum	Mean	SD	CV, %
BW at 1 day of age (g)	146	10.6	6.7	8.5	0.83	9.77
BW at the age of 56 days (g)	146	299.00	169.00	239.19	24.99	10.45
Average daily BW gain (g)	146	6.5	3.8	5.1	0.68	13.32
Dressed weight (g)	146	221.27	119.44	179.59	19.32	10.76
Breast weight (g)	146	93.17	43.21	67.60	9.37	13.86
Thigh weight (g)	146	16.53	7.37	11.36	1.63	14.36
Drumstick weight (g)	146	11.43	4.17	7.94	1.13	14.25

^1^ *n*, number of individuals; BW, body weight; SD, standard deviation; CV, coefficient of variation.

**Table 3:** Chromosome distribution of significant SNPs associated with fattening ability and carcass traits in F_2_ male quails.

	No. of SNPs	Chromosomes
BW ^1^ at 1 day of age	58	1, 2, 4, 5, 6, 8, 9, 13–15, 17, 25, 28
BW at 56 days of age	18	1, 2, 3, 4, 8, 12, 14, 17, 18
Average daily BW gain	8	3, 8, 14, 18
Dressed weight	33	1, 3–5, 7–10
Breast weight	18	1–3, 5, 9, 14, 25
Thigh weight	8	8, 17
Drumstick weight	2	1, 19

^1^ BW, body weight.

**Table 4:** Putative SNPs and candidate genes associated with meat performance in F_2_ quails.

Chr ^1^	SNPs	Traits ^2^	Genes	
			at SNP	Near SNP, ±0.2Mb
2	2:87734460	BW56, BrW	*GNAL*	*AFG3LA*, *PRELID3A*, *TUBB6*, *CIDEA*, *IMPA2*
3	3:16057525	BW56, ADBWG, DCW	–	*DUSP10*, *HLX*, *MTARC2*
8	8:25443595	BW56, DCW *, ADBWG, BrW, TW	–	*CACHD1*, *JAK1*, *DNAJC6*, *LEPR*, *PDE4B*
8	8:25460692	BW56, ADBWG *, DCW *, BrW, TW	*DNAJC6*	*CACHD1*, *RAVER2*, *JAK1*, *LEPR*, *PDE4B*
8	8:25524051	BW56 *, ADBWG *, DCW *, TW *	*LEPR*	*CACHD1*, *RAVER2*, *JAK1*, *DNAJC6*, *PDE4B*, *SGIP1*, *DYNLT5*
8	8:25548447	BW56 *, ADBWG, DCW *, TW *	–	*CACHD1*, *RAVER2*, *JAK1*, *DNAJC6*, *LEPR*, *PDE4B*, *SGIP1*, *MIER1*, *DNA14*, *DYNLT5*
8	8:25548450	BW56 *, ADBWG, DCW *, TW *	–	*CACHD1*, *RAVER2*, *JAK1*, *DNAJC6*, *LEPR*, *PDE4B*, *SGIP1*, *MIER1*, *DNA14*, *DYNLT5*
8	8:25565668	BW56, ADBWG, DCW, TW	–	*CACHD1*, *RAVER2*, *JAK1*, *DNAJC6*, *LEPR*, *PDE4B*, *SGIP1*, *MIER1*, *DNA14*, *DYNLT5*
9	9:19667846	BrW *, DCW	–	*OTOL1*, *SPTSSB*, *NMD3*, *PPM1L*, *B3GALNT1*, *KPNA4*, *TRIM59*, *IFT80*, *SCHIP1*, *IL12A*, *IQCJ*
14	14:8432578	BW56, ADBWG	–	*GDE1*, *CCP110*, *MOSMO*, *VWA3A*, *SDR42E2*, *EEF2K*, *POLR3E*, *CDR2METTL9*, *OTOA*, *IGSF6*, *KDELR2*, *RAC1*, *DAGLB*, *CYTH3*, *RMI2*, *SOCS1*, *CLEC16A*
17	17:3724688	BW56, TW	*SLC27A4*	*SLC25A25*, *NAIF1*, *EEIG1*, *AK1*, *ST6GALNAC6*, *FPGS*, *SH2D3C*, *DPM2*, *ENG*, *CDK9*, *ST6GALNAC4*, *TOR2A*, *URM1*, *CERCAM*, *TRUB2*, *SWI5*, *COQ4*, *DNM1*, *CI21*, *BBLN*, *ODF2*, *GLE1*, *PTGES2*, *SPTAN1*, *DYNC212*
18	18:8756728	BW56, ADBWG	*SPAG9*	*CA10*, *UTP18*, *MBTD1*, *NME1*, *NM9*, *TOB1*, *WFIKKN2*, *LUC7L3*, *ANKPD40*, *CACNA1G*

^1^ Chr, chromosome; ^2^ BW56, body weight at 56 days of age; ADBWG, average daily body weight gain; DCW, dressed weight; BrW, breast weight; TW, thigh weight; DW, drumstick weight. * SNP significance threshold at *p* < 5.4 × 10^−7^.

## Data Availability

The sequence data are accessible to readers by request.

## Supplementary Materials

The following supporting information can be downloaded at: https://www.mdpi.com/article/10.3390/genes15030294/s1, Supplementary Information: Traits and SNPs; Figure S1: Principal component analysis (PCA) for the F_2_ quail reference population based on the GBS data; Figure S2: Quantile–quantile (Q–Q) plots of the GWAS results for the studied meat production traits.
