# Supplementary material for: Genome-Wide Association Study Revealed Putative SNPs and Candidate Genes Associated with Growth and Meat Traits in Japanese Quail
Source: Genes (Basel). 2024 Feb 25;15(3):294. doi: 10.3390/genes15030294 (PMC10970133; doi:10.3390/genes15030294)
Supplement: Supplementary file 1 [file genes-15-00294-s001.zip › Supplementary Figure S1.pdf]

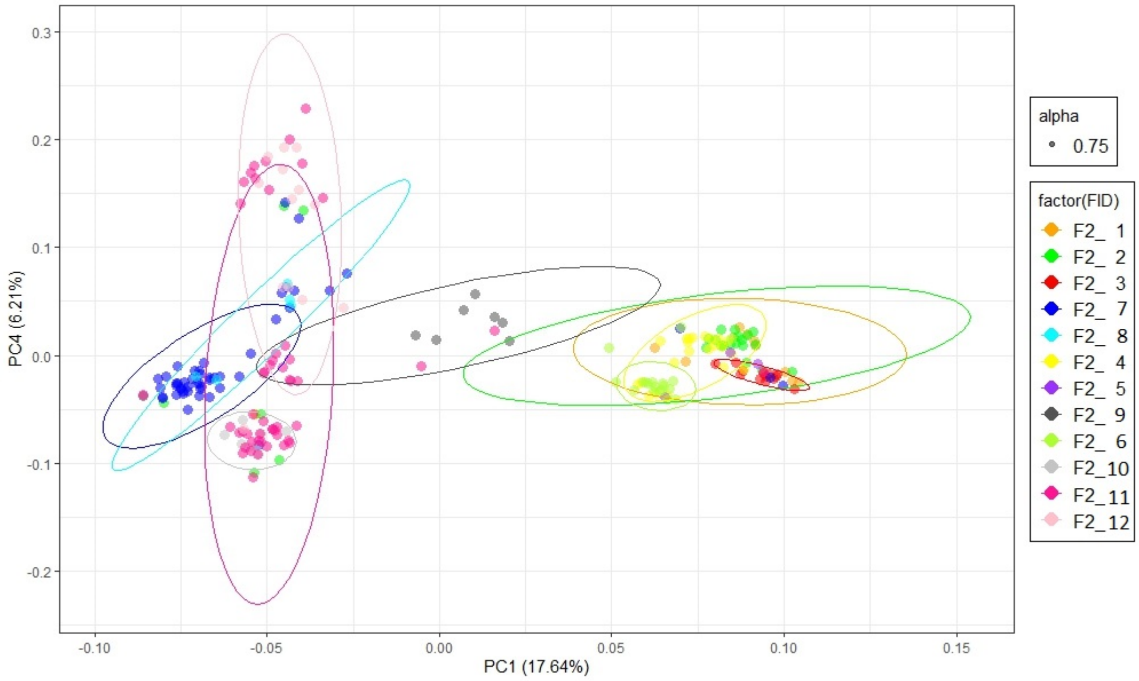

Supplementary Figure S1. Principal component analysis (PCA) for the F_2 quail reference population based on the GBS data. PCA was performed in the plane of the first (PC1, *X*-axis) and fourth (PC4, *Y*-axis) components. Individuals from different groups are indicated by different colors.
