# Supplementary material for: Genome-Wide Association Study Revealed Putative SNPs and Candidate Genes Associated with Growth and Meat Traits in Japanese Quail
Source: Genes (Basel). 2024 Feb 25;15(3):294. doi: 10.3390/genes15030294 (PMC10970133; doi:10.3390/genes15030294)
Supplement: Supplementary file 1 [file genes-15-00294-s001.zip › Supplementary Figure S2.pdf]

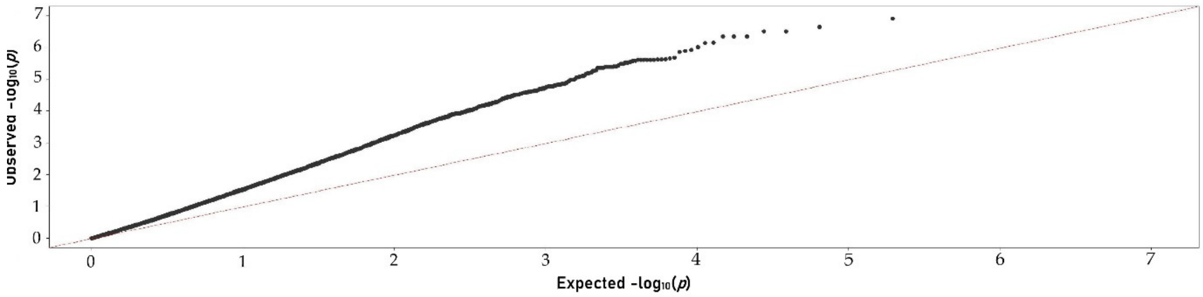

Observed -log10(p)
Expected -log10(p)

A

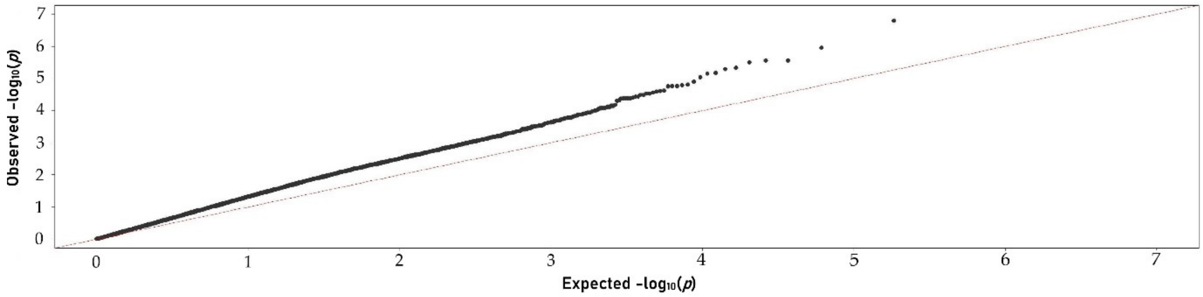

Observed -log10(p)
Expected -log10(p)

B

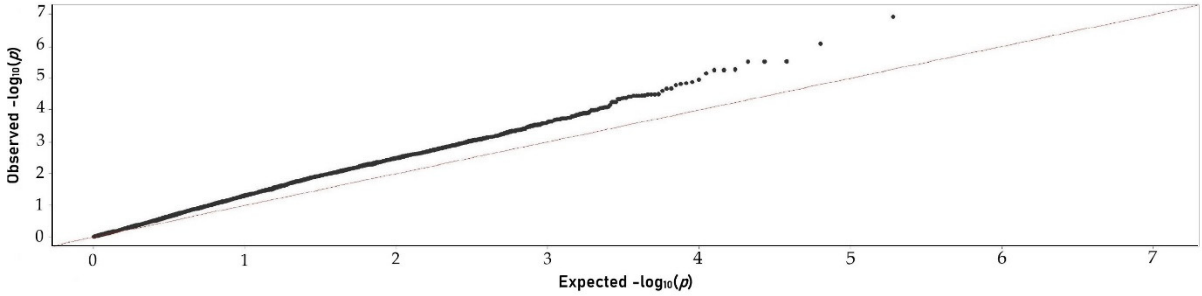

Observed -log10(p)
Expected -log10(p)

C

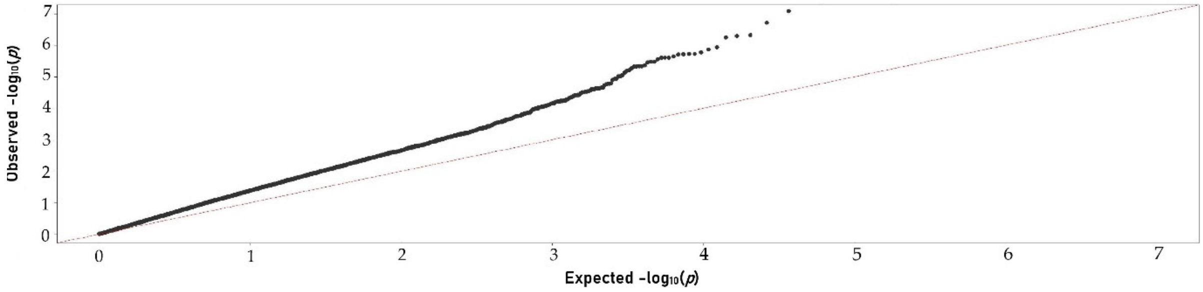

Observed -log10(p)
Expected -log10(p)

D

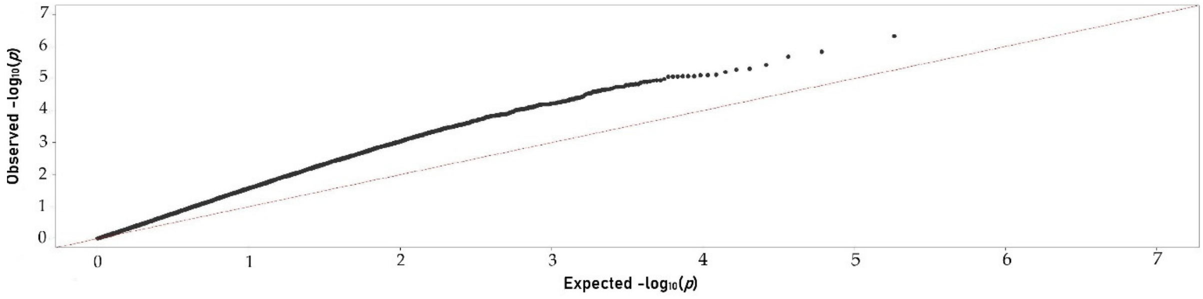

Observed -log10(p)
Expected -log10(p)

E

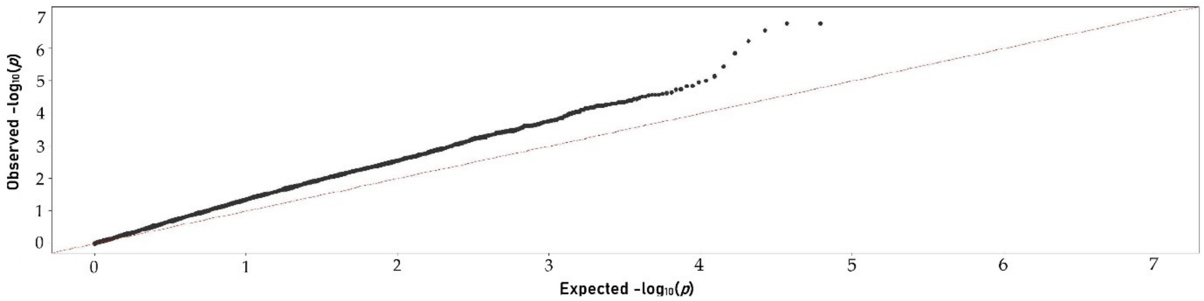

F

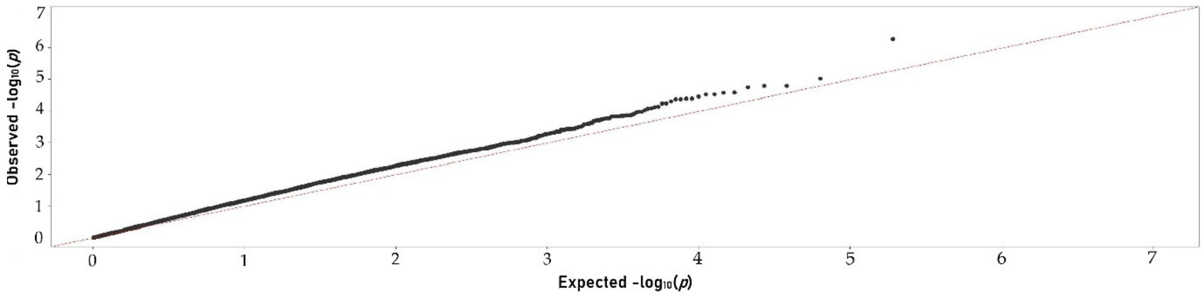

G

Supplementary Figure S2. Quantile–quantile (Q–Q) plots of the GWAS results for the studied growth and meat production traits: (**A**) body weight at 1 day of age, (**B**) body weight at 56 days of age, (**C**) average daily body weight gain, (**D**) dressed carcass weight, (**E**) breast weight, (**F**) thigh weight, and (**G**) drumstick weight. Q–Q plots represented quantiles of the probability distribution of expected and observed deviations from the normal distribution for nominal significance values (−log10 (*p*)).
